# Meta-analysis of diagnostic performance of serology tests for COVID-19: impact of assay design and post-symptom-onset intervals

**DOI:** 10.1080/22221751.2020.1826362

**Published:** 2020-10-07

**Authors:** Hongyu Wang, Jingwen Ai, Michael J. Loeffelholz, Yi-Wei Tang, Wenhong Zhang

**Affiliations:** a Department of Infectious Diseases, Huashan Hospital, Shanghai Medical College, Fudan University, Shanghai, People’s Republic of China; b Cepheid China, Danaher Diagnostic Platform, Shanghai, People’s Republic of China

**Keywords:** COVID-19, SARS-CoV-2, serology, immunoassays, metanalysis

## Abstract

Serology detection is recognized for its sensitivity in convalescent patients with COVID-19, in comparison with nucleic acid amplification tests (NAATs). This article aimed to evaluate the diagnostic accuracy of serologic methods for COVID-19 based on assay design and post-symptom-onset intervals. Two authors independently searched PubMed, Cochrane library, Ovid, EBSCO for case–control, longitudinal and cohort studies that determined the diagnostic accuracy of serology tests in comparison with NAATs in COVID-19 cases and used QUADAS-2 for quality assessment. Pooled accuracy was analysed using INLA method. A total of 27 studies were included in this meta-analysis, with 4 cohort, 16 case–control and 7 longitudinal studies and 4565 participants. Serology tests had the lowest sensitivity at 0–7 days after symptom onset and the highest at >14 days. TAB had a better sensitivity than IgG or IgM only. Using combined nucleocapsid (N) and spike(S) protein had a better sensitivity compared to N or S protein only. Lateral flow immunoassay (LFIA) had a lower sensitivity than enzyme-linked immunoassay (ELISA) and chemiluminescent immunoassay (CLIA). Serology tests will play an important role in the clinical diagnosis for later stage COVID-19 patients. ELISA tests, detecting TAB or targeting combined N and S proteins had a higher diagnostic sensitivity compared to other methods.

## Introduction

On 11 March 2020, the World Health Organization (WHO) described the global COVID-19 outbreak as a worldwide pandemic^1^. SARS-CoV-2 is the etiologic agent of COVID-19 and primarily attacks the human respiratory system and can cause respiratory infections, diarrohea, and even multiple organ failure in patients^2^. By 10 July 2020, there were 12,102,328 cases of COVID-19 diagnosed worldwide and 551,046 deaths had been reported^3^. At the time of writing, the pandemic was still severe and the likelihood of persistence of SARS-CoV-2 within the human population is increasing.

As no definitely effective drugs or vaccines are yet available, rapid diagnosis of SARS-CoV-2 infection and quick isolation of the patients and tracing of their close contacts are currently the most effective means of preventing transmission. At present, the definitive diagnosis of COVID-19 mainly depends on the detection of SARS-CoV-2 RNA by nucleic acid amplification tests (NAATs) such as RT-PCR^4^. Serological methods have also become an important auxiliary testing tool, and play an important role in the diagnosis and epidemiological investigation of COVID-19 cases^5–10^. At the time of writing, the United States Food and Drug Administration has granted Emergency Use Authorization for 31 serology test kits^11^. Serological test methods for the detection of anti-SARS-CoV-2 IgG and IgM antibody include enzyme-linked immunosorbent assay (ELISA), chemiluminescent immunoassay (CLIA), and lateral flow immunoassay (LFIA).

Compared with some NAATs, serological testing is relatively easier to perform and requires less technologically advanced equipment. In addition, the blood samples are less likely to contain infectious SARS-CoV-2 virus than respiratory specimens, decreasing the potential risk of infection to laboratory staff^12^. However, there are questions remaining to be answered concerning the serological diagnosis of COVID-19. First, studies have reported that the seroconversion happened at 3–14 days post symptom onsets^13,14^, which may not facilitate the early diagnosis of the disease. What’s more, the window periods of the different serological tests have not been directly assessed. Second, the specificity and sensitivity of serological methods can vary over the infection time course, and need to be further analysed^15^. Finally, the impact of assay design on the performance of serological tests has yet to be determined.

Meta-analysis is a quantitative evaluation method in evidence-based medicine and is widely accepted as one of the most reliable tools in clinical analysis. Our study evaluated all published case–control, longitudinal and cohort studies for the diagnostic efficacy and characteristics of the current serological tests for COVID-19.

## Materials and methods

### Selection criteria

The inclusion criteria for this meta-analysis were the following: (1) all cohort, case–control, and longitudinal studies published between 1 January 2020 and 30 June 2020; (2) all studies that evaluated the diagnostic performance of serological tests for COIVD-19 in comparison with a SARS-CoV-2 NAAT as a reference test; (3) studies from which we could directly or indirectly extract data on true positives (TP), false positives (FP), false negatives (FN), and true negatives (TN); (4) participants were 18–85 years of age; (5) published articles as well as letters and corrected proofs; and (6) only articles in English were included.

The exclusion criteria were the following: (1) preprint articles which had not been peer reviewed; (2) studies that had crossed data with other published articles; (3) participants were immunocompromised (cancer, AIDS patients, etc.); and (4) studies published before 2020. (5) Studies with more than one “high risk of bias” in QUADAS-2 quality assessment domain 2–4 were excluded.

### Search strategy

We searched the databases using the following Medical Subject Heading words and key words, or the combination: COVID-19, SARS-CoV-2, severe acute respiratory syndrome coronavirus 2, serology, serology test, antibody, antigen, diagnostic test. Main medical databases including PubMed, Cochrane library, EBSCO, and OVID were searched in this study (Full search strategy in supplementary material (1). We set a time limit published between 1 January 2020 and 30 June 2020 and a language limit of English only.

### Study evaluation and data extraction

Two researchers (Wang and Ai) independently scrutinized abstracts and titles to include potentially eligible articles and acquire full texts online. Articles unavailable online were excluded. Then, the same two researchers examined the full texts individually using the preset inclusion and exclusion criteria.

As recommended by Cochrane Handbook for Systematic Reviews of Diagnostic Accuracy^16^, we adopted QUADAS-2 (Quality Assessment of Diagnostic Accuracy Studies -2) to evaluate the bias and quality of selected studies^17^. The following four domains were considered for risks of bias and application concerns as depicted in the assessment tool: (1) participant selection; (2) index test; (3) reference text; and (4) flowing and timing. Studies with more than one “high risk of bias” in the later 3 part were excluded (supplementary material 2).

The following information was extracted from final eligible studies: (1) details of the study: author, title, published date, countries where studies were conducted, study design, participant inclusion manner and criteria, number of enrolled participants and the grouping, number of participants whose results were available; (2) clinical characteristics of participants: age, gender, COVID-19 status; (3) target data: the results of serologic tests and NAATs for COVID-19 (TP, FP, FN, TN) and symptom onset-specimen collection interval (days). One sample per participant was included in the overall sensitivity and specificity, while the accuracy on different post-symptom interval directly used the respective data from the articles; and (4) test profile: methods for serology and SARS-CoV-2 RNA detection, profile of detected antibodies, and targeted antigen of serologic tests.

### Statistical analysis

We assessed risks of bias and application concerns using QUADAS-2 tool on Review Manager 5.4 software^18^. Meta-analysis over selected studies was performed using R software (version 3.6.1) with the meta4diag package^19^. TAB was defined as combined IgG and IgM results, or directly described in the primary articles. Diagnostic performance of IgG, IgM, TAB (or combined IgG and IgM), were analysed. Sensitivity and specificity were calculated. Data synthesis was performed using Bayesian bivariate integrated nested Laplace approximation (INLA) method according to the protocol^19^. Forest plots of point estimates and 95% confidence intervals (95% CI) were provided. Summary receiver-operating characteristic (SROC) curves were plotted to evaluate the heterogeneity (threshold effect) between studies^20^.

## Result

### Search results

**Figure 1.**
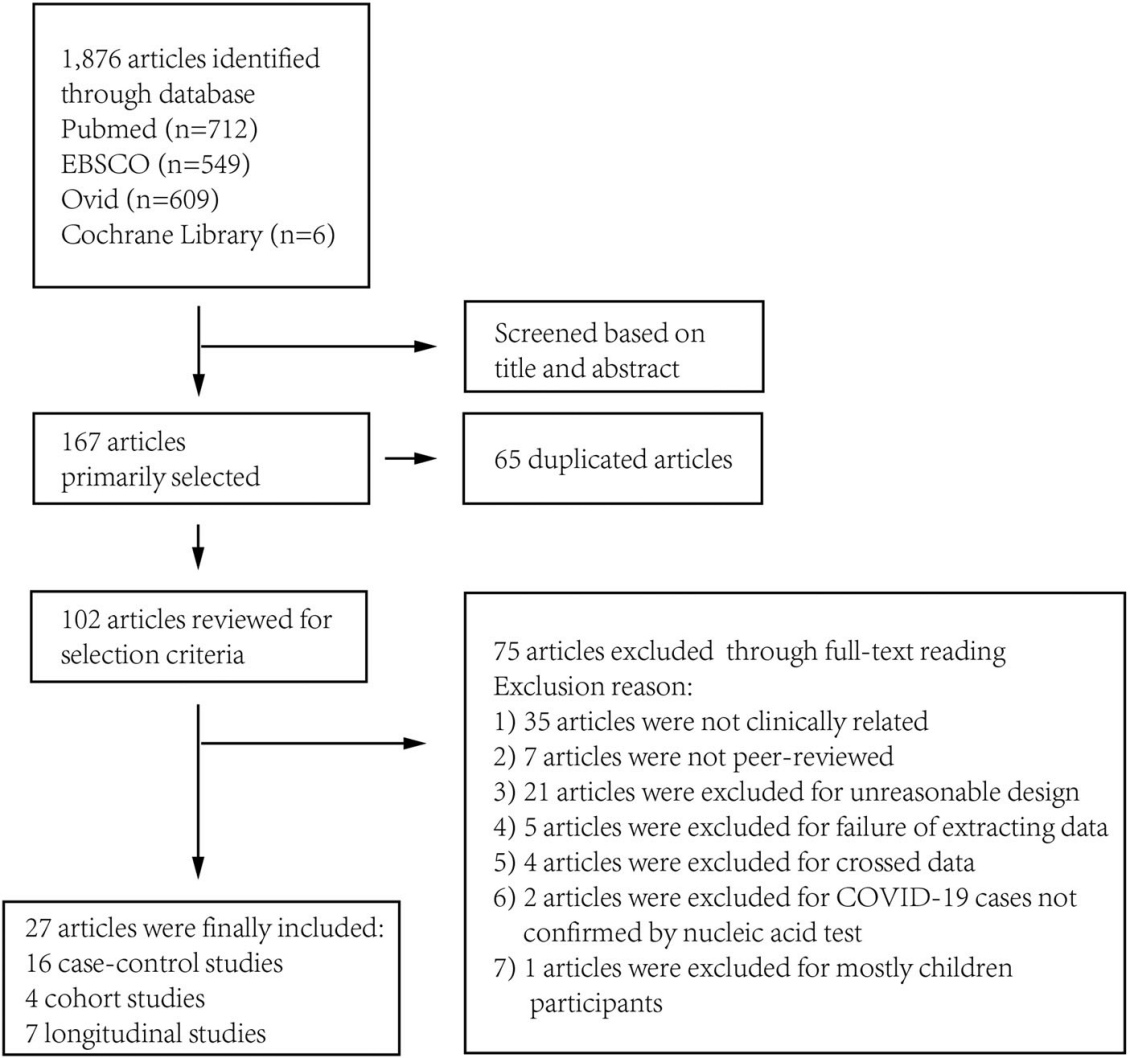
Search process of the meta-analysis.

A total of 1876 articles were identified by systematic literature research as of30 June 2020. A total of 167 studies were selected through title and abstract, in which 65 were duplicated and 102 were selected for further review. Through full-text review, 75 articles were excluded as depicted in Figure 1 and supplementary Table 1. A total of 27 articles were finally included for analysis: 16 case–control studies; 7 longitudinal studies; and 4 cohort studies^21–47^.

**Figure 2.**
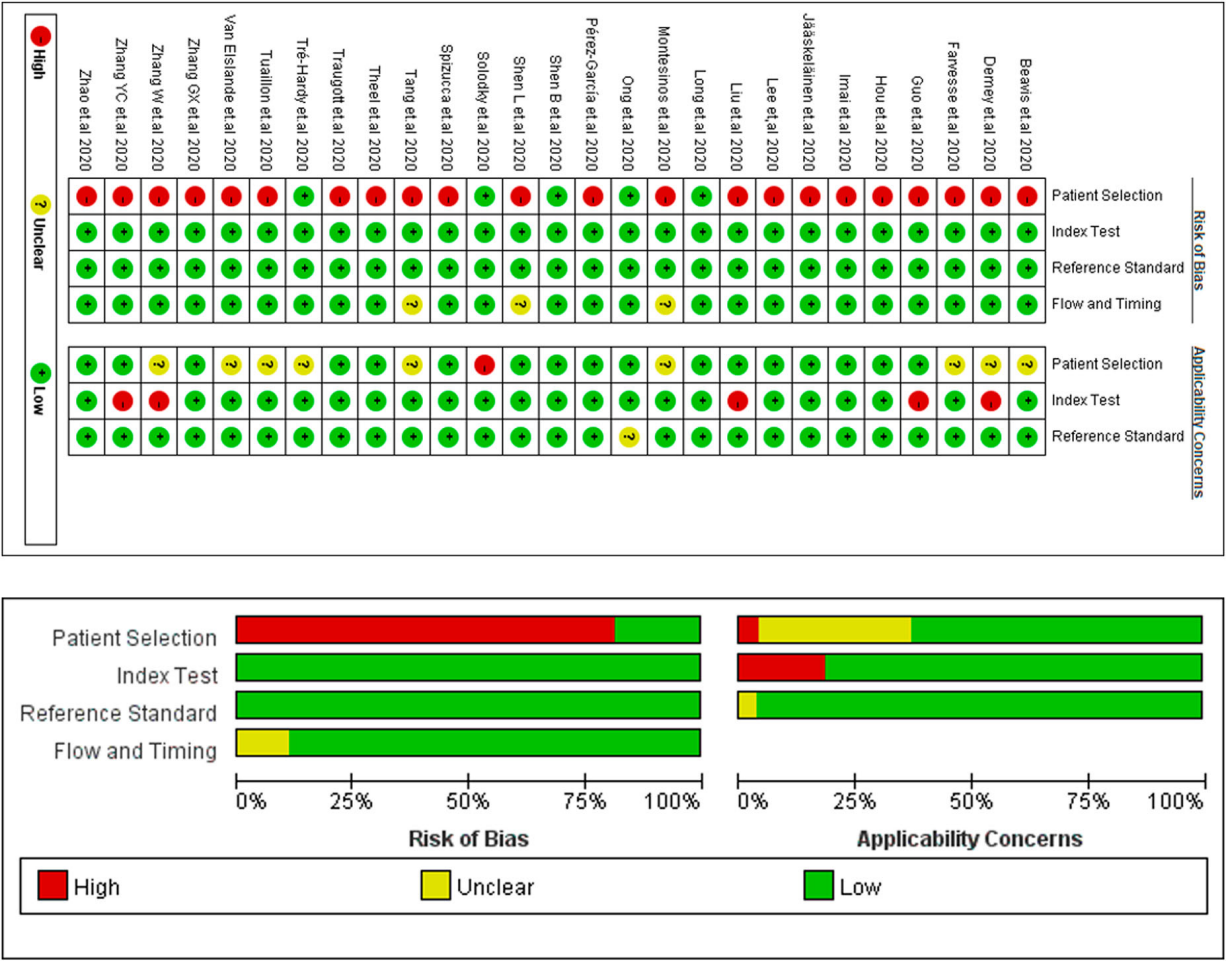
Risk of bias and application concerns of included studies assessed using QUADAS-2 tool. Red spots refer to high risk of bias or high concern, yellow refer to unclear and green refer to low.

Assessment of risks of bias and application concerns are described in Figure 2. 85.1% (23/27) studies were present with a high risk of bias in patient selection, where these articles did not avoid case–control or longitudinal design. We involved these studies for later analysis and evaluated possible risks of bias in discussion.

Detailed characteristics of these 27 articles are shown in Table 1. A total of 4565 subjects were included for analysis. 37.0% (10/27) of the studies were conducted in China. 13, 6, 8, 9 studies performed ELISA, CLIA and LFIA for serology test, respectively. 77.8% (21/27) studies performed a serology test that targeted S protein/receptor binding domain (RBD) protein or N protein of COVID-19 virus.

**Table 1.** Characteristics of studies included in the meta-analysis of serology test diagnostic performance.

ID	Nation	Study Design	Included/Total Subjects (N)	Included Subject Distribution	Kit Company	Method	Item	Antigen	Age*	Male (%)*	Severe cases (%)*	Reason for not full inclusion
Hou et al. 2020^21^	Wuhan, China	Longitudinal	338/338	338 COVID-19 cases	YHLO Biotech Co. Ltd. Shenzhen, China	CLIA	IgG/IgM	nucleoprotein/spike protein	63.3	50.6%	22.2%	-
Long et al. 2020^22^	Chongqing, China	Part 1: Case-Control Part 2: Cohort Study	478/501	262 COVID-19 cases 52 RT-PCR (-) suspected cases 164 close contacts	Bioscience Co., Ltd, Chongqing, China	CLIA	IgG/IgM	nucleoprotein/spike protein	47	55.4%	13.7%	23 cases with unclear records of symptom onset
Solodky et al. 2020^23^	France	Cohort Study	244/329	244 health centre workers	TODA Pharma, Strasbourg, France	LFIA	IgG/IgM	unclear	-	-	-	85 cases excluded were cancer patients, with abnormal immune response
Zhao et al. 2020^24^	Shenzhen, China	Longitudinal	173/173	173 COVID-19 cases	Wantai Biological Pharmacy Enterprise Co., Ltd., Beijing, China	ELISA	IgG/IgM	nucleoprotein	48	48.6%	18.5%	-
Guo et al. 2020^25^	Beijing, China	Case-Control	425/425	82 COVID-19 cases 58 RT-PCT (-) suspected cases 135 cases before 2020 150 healthy blood donors before 2020	Self-produced	ELISA	IgG/IgM	nucleoprotein	-	-	34.1%	-
Tang et al. 2020^26^	USA	Case-Control	201/201	48 COVID-19 cases 80 RT-PCT (-) suspected cases 50 cases before 2020 23 other infection in 2020	EUROIMMUN, Lubeck, Germany et.al	ELISA	IgG	nucleoprotein/spike protein	-	-	-	-
Farvesse et al. 2020^27^	Belgium	Case-Control	176/176	97 COVID-19 cases 79 cases before 2020	Hoffmann-La Roche Co., Ltd., Rotkreuz, Switzerland	CLIA	Tab	nucleoprotein	-	-	-	-
Van Elslande et al. 2020^28^	Belgium	Case-Control	201/201	94 COVID-19 cases 103 cases before 2020	EUROIMMUN, Lubeck, Germany et.al	ELISA LFIA	IgG/IgM	spike protein unclear	67.5	70.2%	30.9%	-
Ong et al. 2020^29^	Utrecht	Cohort Study	228/228	99 COVID-19 cases 129 RT-PCR (-) suspected cases	Orient Gene Biotech Co., Ltd., Zhejiang, China et.al	ELISA LFIA	IgG/IgM	unclear spike protein	61	52.0%	9.0%	-
Liu et al. 2020^30^	Chongqing, China	Longitudinal	32/32	32 COVID-19 cases	Xinsaiya Biotechnology Co., Ltd., Chongqing, China	Unclear	IgG/IgM	spike protein	55	66.7%	56.3%	-
Shen L et al. 2020^31^	Xiangyang, China	Case-Control	188/188	103 COVID-19 cases 25 RT-PCR (-) suspected cases 10 other diseases in 2020 50 health donors before 2020	Outdo Biotech Co., Ltd., Shanghai, China	LFIA	IgM	nucleoprotein/spike protein	25	45.0%	13.6%	-
Zhang YC et al. 2020^32^	Nanjing, China	Longitudinal	21/21	21 COVID-19 cases	Innovita Co., Ltd., Beijing, China	LFIA	IgG/IgM	nucleoprotein/spike protein	37	61.9%	23.8%	-
Demey et al. 2020^33^	France	Case-Control	34/34	22 COVID-19 cases 12 cases before 2020	ISIA BIO-Technology Co., Ltd, Chongqing, China et.al	LFIA	IgG/IgM	unclear	-	-	-	-
Zhang W et al. 2020^34^	Wuhan, China	Longitudinal	16/16	16 COVID-19 cases	Kyab Biotech Co., Ltd, Wuhan, China	ELISA	IgG/IgM	nucleoprotein	-	-	18.8%	-
Tuaillon et al. 2020^35^	France	Case-Control	58/58	38 COVID-19 cases 20 cases before 2020	EUROIMMUN, Lubeck, Germany et.al	ELISA LFIA	IgG/IgM	nucleoprotein/spike protein	67	57.9%	68.4%	-
Spizucca et al. 2020^36^	Italy	Case-Control	37/37	23 COVID-19 cases 7 RT-PCR (-) suspected cases 7 RT-PCR (-) asymptomatic controls	Diagreat Biotechnologies Co., Ltd, Beijing, China	LFIA	IgG/IgM	unclear	57	-	52.2%	-
Lee et al. 2020^37^	Taiwan, China	Longitudinal	42/42	14 COVID-19 cases 28 RT-PCT (-) controls	ALLTEST Biotech Co., Ltd. Hangzhou, China	LFIA	IgG/IgM	nucleoprotein	51	50.0%	42.9%	-
Theel et al. 2020^38^	USA	Case-Control	205/310	56 COVID-19 cases 149 health donors before 2020	EUROIMMUN, Lubeck, Germany et.al	ELISA CLIA CMIA	IgG	nucleoprotein/spike protein	51	53.6%	-	105 cases in early 2020 were not tested by RT-PCR for COVID-19
Traugott et al. 2020^39^	Austria	Case-Control	177/177	77 COVID-19 cases 60 RT-PCR (-) controls 40 cases before 2020	Euroimmun, Lübeck, Germany et.al	ELISA LFIA	IgG/IgM	spike protein unclear	63	62.3%	-	-
Shen B et al. 2020^40^	Taizhou, China	Cohort study	150/150	150 suspected COVID-19 cases	Outdo Biotech Co. Ltd, Shanghai, China	LFIA	IgG/IgM	unclear	40	59.3%	21.6%	-
Beavis et al. 2020^41^	USA	Case-Control	150/178	64 COVID-10 cases 70 RT-PCR (-) controls 16 cases before 2020	Euroimmun, Lübeck, Germany	ELISA	IgG	nucleoprotein	-	-	-	28 cases in 2020 not tested by RT-PCR for COVID-19
lmai et al. 2020^42^	Japan	Case-Control	160/160	112 COVID-19 cases 48 cases before 2020	Artron, Burnaby, Canada	LFIA	IgG/IgM	unclear	67	57.1%	-	-
Montesinos et al. 2020^43^	Belgium	Case-Control	200/200	128 COVID-19 cases 62 cases before 2020 10 health donors in 2020	Euroimmun, Luebeck, Germany et.al	ELISA CLIA LFIA	IgG/IgM	spike protein/ABEI	-	-	-	-
Tré-Hardy et al. 2020^44^	Belgium	Cohort Study	125/125	125 clinically suspected COVID-19 cases	Euroimmun, Lübeck, Germany et.al	ELISA CLIA	IgG	nucleoprotein/spike protein	-	-	-	-
Zhang GX et al. 2020^45^	Wuhan, China	Longitudinal	112/112	112 COVID-19 cases	Yahuilong Biotechnology, Shenzhen, China	Unclear	IgG/IgM	nuceloprotein/envelop protein	39	29.5%	-	-
Jääskeläinen et al. 2020^46^	Finland	Case-Control	143/143	62 COVID-19 cases 81 cases before 2020	Abbott, Illinois, USA et.al	ELISA LFIA	IgG/IgM	nucleoprotein/spike protein	54	45.9%	28.6%	-
Pérez-García et al. 2020^47^	Spain	Case-Control	251/251	90 COVID-19 cases 61 PCR (-) cases 100 cases before 2020	AllTest Biotech, Hangzhou, China	LFIA	IgG/IgM	unclear	64	57.8%	28.9%	-

*Age, male and severe cases were the mean value or percentage in RNA-confirmed COVID-19 cases.

Abbreviations: RT-PCR: real-time polymerase chain reaction; NAT: nucleic amplification test; ELISA: enzyme linked immune sorbent assay; CLIA: chemiluminescent immunoassay; LFIA: lateral flow (immune)assay.

### Pooled diagnostic performance of IgG, IgM, TAB for COVID-19

**Figure 3.**
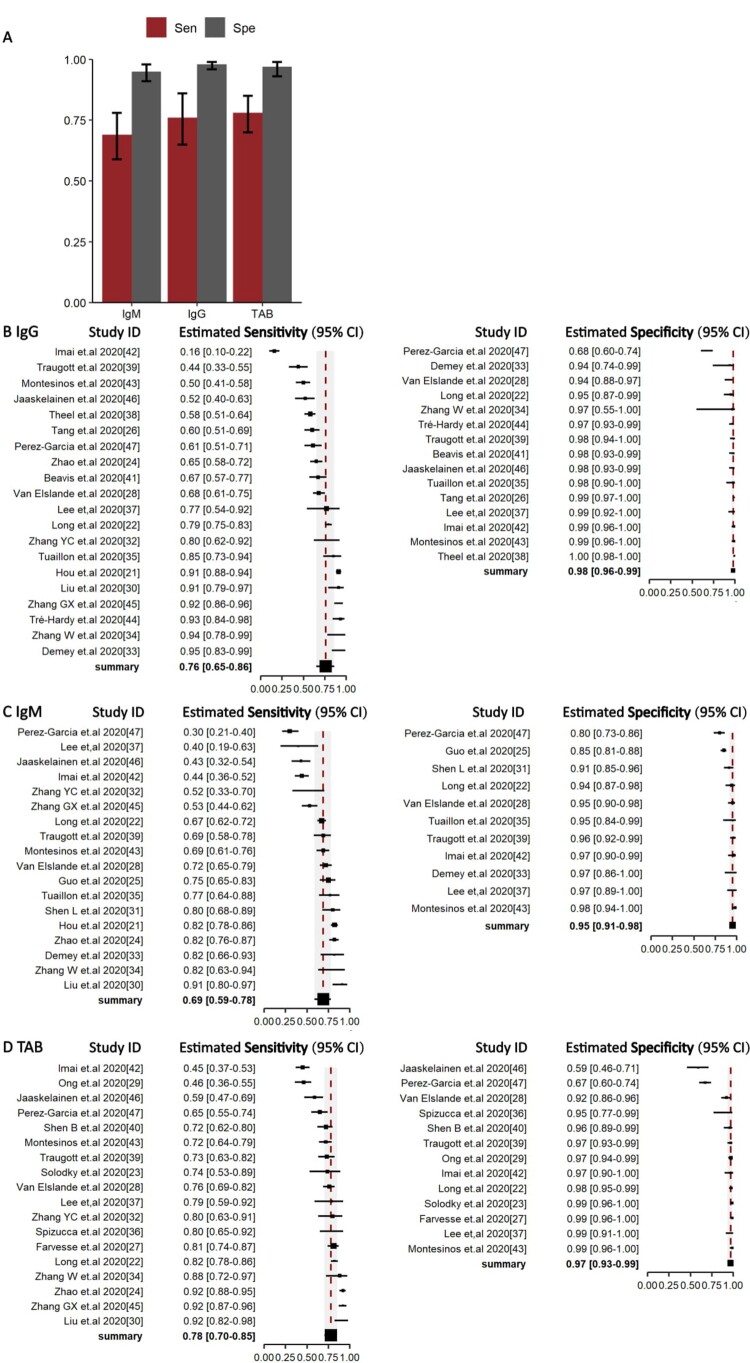
Overall Sensitivity and Specificity of Serology test in NAAT-confirmed COVID-19 cases. (A) Histogram of sensitivity and specificity in IgG, IgM, total antibody. Median (column) and 95% CI (error bar) were shown in the histogram. (B–D) forest plots of sensitivity (Right) and specificity (Left) in IgG, IgM, total antibody. *Abbreviations: TAB: Total antibody*.

**Figure 4.**
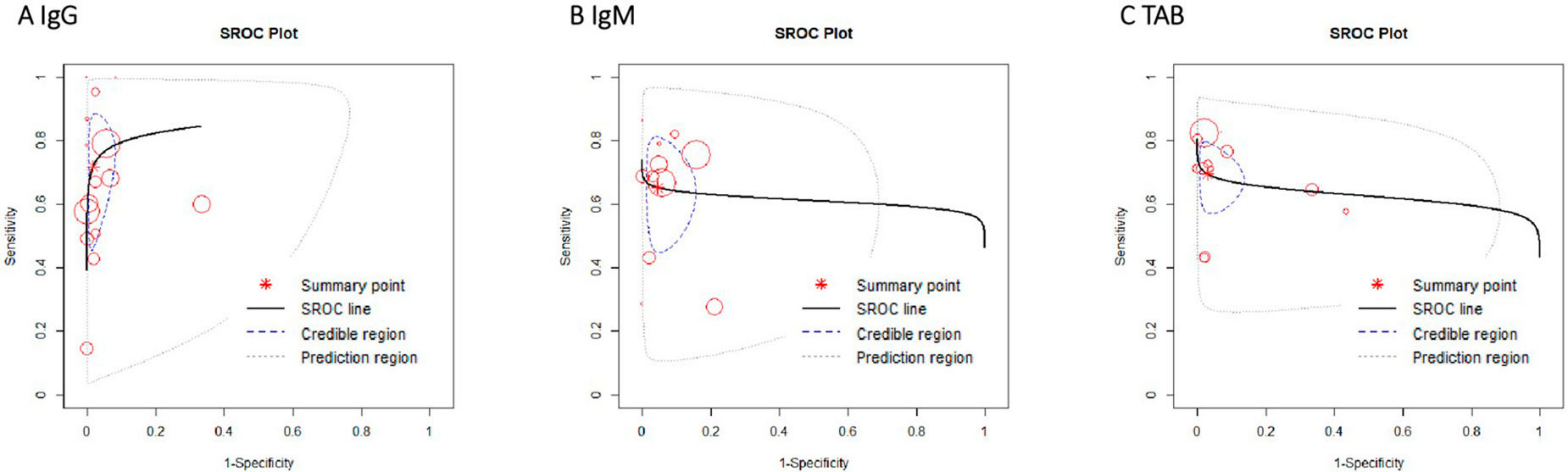
Summary receiver-operating characteristic of IgG (A), IgM (B), TAB (C).

The pooled sensitivity of IgG, IgM, and TAB in RNA-positive COVID-19 cases was 0.76 (95%CI 0.65–0.86), 0.69 (95%CI 0.59–0.78), and 0.78 (95%CI 0.70–0.85) (Figure 3(B–D)), respectively. The specificity of IgG, IgM, and TAB was 0.98 (95%CI 0.96–0.99), 0.95 (95%CI 0.91–0.98), and 0.97 (95%CI 0.93–0.99), respectively (Figure 3(B–D)). There was no heterogeneity between studies (Figure 4).

### Dynamic sensitivity of serologic tests after symptom onset

**Figure 5.**
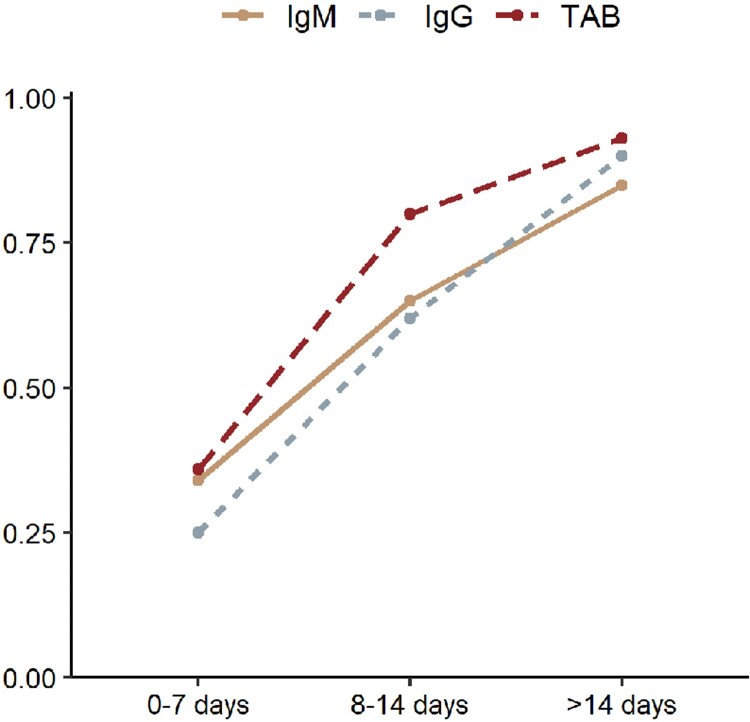
Dynamic change of the sensitivity of serology test at 0–7, 8–14, >14 days since symptom onset.

At 0–7 days, 12, 11, 10 articles were included for pooled analysis of IgG, IgM and TAB. At 7–14 days, 12, 10, 10 articles were included for pooled analysis of IgG, IgM and TAB. At over 14 days, 12, 11, 11 articles were included for pooled analysis of IgG, IgM and TAB. Sensitivity of IgG, IgM and TAB was 0.25 (95%CI 0.16–0.36), 0.34 (95%CI 0.25–0.42), and 0.36 (95%CI 0.28–0.43), respectively during the first 7 days after symptom onset, but increased to 0.62 (95%CI 0.52–0.71), 0.65 (95%CI 0.36–0.86), 0.80 (95%CI 0.69–0.99) at 8–14 days post symptom onset, and 0.90 (95%CI 0.86–0.93), 0.85 (95%CI 0.68–0.95), 0.93 (95%CI 0.80–0.98), respectively after 14 days post symptom onset in comparison with NAATs at diagnosis (Figure 5, supplementary figure).

### Diagnostic performance of different serologic test methods, and by targeted antigen

**Figure 6.**
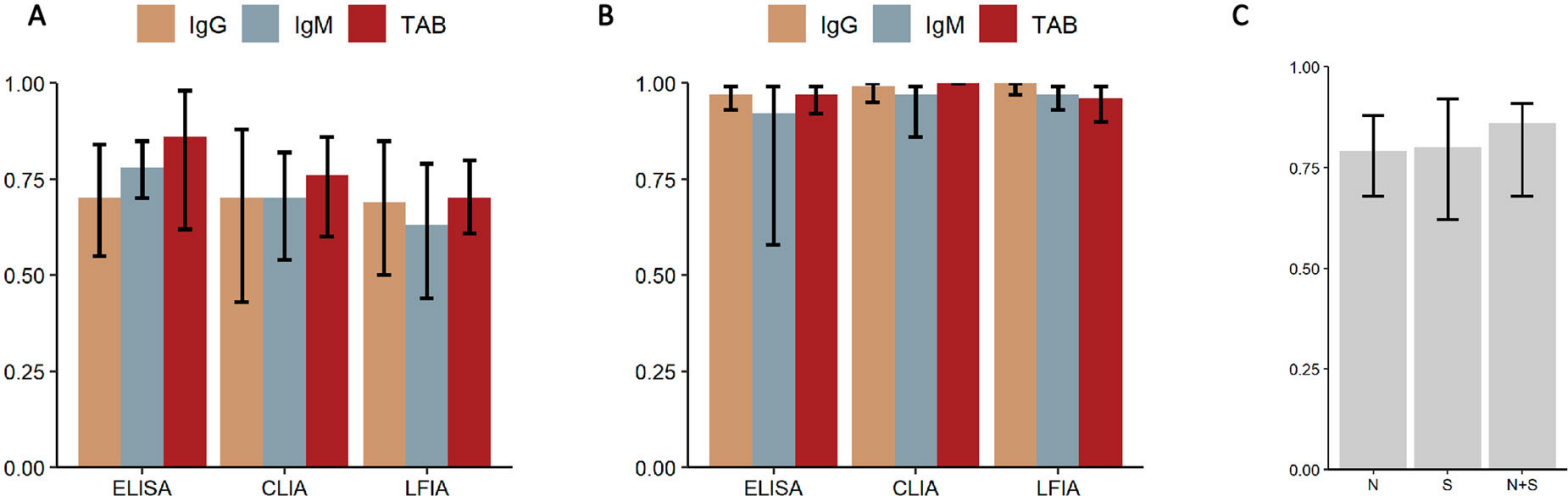
Sensitivity of serology test in different method or targeted antigen. (A) Histogram of the sensitivity of serology test in ELISA, CLIA, and LFIA. (B) Histogram of the specificity of serology test in ELISA, CLIA, and LFIA. (C) Histogram of the sensitivity of serology test when targeted on spike protein (S), nucleoprotein (N) or both (N + S). *Abbreviations: ELISA: Enzyme linked immune sorbent assay; CLIA: Chemiluminescent immunoassay; LFIA: Lateral flow (immuno)assay*.

The sensitivity of different serologic methods is plotted in Figure 6(A). Seven studies provided direct comparison between different methods while 20 articles didn’t (supplementary Table 2). ELISA had the highest sensitivity in IgG, IgM and TAB with estimated sensitivity of 0.70 (95%CI 0.55–0.84), 0.78 (95%CI 0.70–0.85), 0.86 (95%CI 0.62–0.98), respectively. LFIA had the lowest sensitivity in IgG, IgM or TAB, with estimated sensitivity of 0.69 (95%CI 0.5–0.85), 0.63 (95%CI 0.44–0.79), 0.70 (95%CI 0.61–0.80), respectively. Pooled specificity of ELISA, CLIA, LFIA ranged from 92% to 100% (Figure 6(B)). The sensitivity of tests targeting N, S and both (combined) antigens was 0.79 (95%CI 0.68–0.88), 0.80 (95%CI 0.62–0.92), and 0.86 (85%CI 0.68–0.91), respectively (Figure 6(C)).

## Discussion

Our meta-analysis included 27 articles, with 4 cohort studies, 16 case–control studies and 7 longitudinal studies to evaluate the overall diagnostic performance of serology tests for diagnosis of COVID-19, including the optimum time window and best performing methodology. Serology tests had a sensitivity of less than 40% at 0–7 days post symptom onset. Serology tests detecting TAB had a higher sensitivity than IgM or IgM alone. Targeting combined N and S proteins had a higher sensitivity than targeting N or S protein alone. LFIA tended to have a lower sensitivity than ELISA or CLIA.

The overall sensitivity of serology tests was poor, thus negative serological results alone cannot exclude the diagnosis of COVID-19. However, significant variation was observed in the forest plots of the sensitivity of serology tests (Figure 3(B–D)), with a range of 16%–93% in IgG, 42%–92% in IgM and 45%–92% in TAB. We attributed this mostly likely to different seroconversion times for different antibody classes, and further divided included articles according to symptom onset-specimen collection interval^48^. Our analysis suggested that serology tests had the lowest sensitivity at 0–7 days post symptom onset and the highest sensitivity at >14 days. Our findings and those of others suggest that 14 days post symptom onset is a point when the sensitivity serology tests is sufficiently high to replace NAATs for the optimal diagnosis of COVID-19^13,49–52^. During the early acute phase of infection, antibody detection might cause numerous false negatives cases. Nonetheless, there have been rare detectable antibody responses during the early phase of COVID-19 concurrent with high virus load and a high risk of transmission^53^. In the late phase of disease, on the contrary, seroconversion occurs when virus load begins to decline, and serological tests might play a more important role in the diagnosis of COVID-19. Overall, our pooled analysis suggests a preferred diagnostic algorithm based on days post symptom onset: NAAT alone at 0–14 days, NAAT combined with a serology test at over 14 days, when virus shedding might drop below the detection limit of most NAATs^54^.

As for the serology test methodology, our analysis suggested that serology tests detecting TAB (or combined IgG and IgM), targeting N and S combined may provide greater sensitivity than tests based on N or S alone. LFIA had a relatively low sensitivity than ELISA or CLIA but provided a fast turn-around time and convenience, and had been authorized by FDA for emergency use. The choice of serology test methodology should be based on testing environment and patient population. LFIA tests could prove useful in the emergency room, ambulatory and outpatient settings rather than simply abandoned for its relatively poor performance. We didn’t pool our analysis based on assays from different companies, but other head-to-head studies had shown a variable accordance between different assays within only a small group of participants^28,38,46,55^. A recent study showed a high accordance between Abbott Architect, DiaSorin Liaison, Ortho VITROS, and Euroimmun among 1200 serum samples^56^. Considering that the clinical performance of commercial assays was varied from laboratory condition, immune status of participants, time from symptoms onset to sample collection, etc., more head-to-head comparison was needed to figure out the accordance between commercial assays on a relatively larger scale.

In this study, most studies remained to had no risk of bias in the domain 2–4 or fewer application concern compared with other meta-analysis of diagnostic test accuracy. We attributed this phenomenon due to the following reasons. First, studies with high risk of bias in the domain 2–4 were excluded. The detailed exclusion reasons included no prespecified threshold for serology test, not using NAATs as reference tests, not all participants receiving the NAATs, etc. All of these problems were considered to bring high risk of concerns while the first domain, with a non-cohort study design or unclear consecutive enrolment were considered to bring less effect to the analysis. Second, COVID-19 was a global public health problem broke out within less than one year and thus studies on serology test accuracy of COVID-19 had some similar features: (1) Participant enrolment was confined to a short time and the criterion was usually not complex, with no clear exclusion criterion. (2) NAATs is the only method suggested by WHO to diagnose COVID-19. (3) Most case–control studies used preserved serum or blood before 2020 as the control group for determining the accuracy for serology test. These features also led to a high agreement between enrolled articles in the assessment of risk of bias and application concern using QUADAS-2 tool.

Previously, NAATs were the recommended gold standard for COVID-19 diagnosis by the WHO, while antigen tests were not recommended due to insufficient performance data^57,58^. Another concern raised by the WHO regarding serology tests was the relatively long antibody window, with seroconversion occurring during the second week after symptom onset^52^. At present, antibody detection was only suggested for epidemiological research or disease surveillance^5,9,59,60^. This is the first study that meta-analysed the sensitivity of serology tests across different time windows. It also provides a general review of different serology test methods. Combined IgG and IgM, as well as combined N and S protein-based tests had better performance than IgG/IgM alone, or N/S protein alone based tests, while among method formats, LFIA had lower sensitivity than ELISA or CLIA.

This study has some limitations. First, we did not analyse the cross-reactivity/specificity of serology tests for COVID-19. This was limited by data extraction, where most qualified articles did not provide specificity data. Previous studies had reported that the serological cross-reactivity between COVID-19 and other coronavirus disease like SARS-CoV seemed to be high, suggesting that serology tests might bring more false negativities and should only be applied as a supplementary tool for clinical diagnosis^61^. Second, 23/27 (85.2%) of enrolled articles were present with high risks of bias for case–control or longitudinal design. Specificity in our study might be overestimated because most of the control group used samples from healthy donors before 2020, which avoid possible cross-reactivities as mentioned above. Another limitation was that we did not analyse the combined diagnostic performance of NAATs and serology tests, because clinically confirmed COVID-19 cases without positive RNA or serology test results were not enrolled into this meta-analysis. According to our study, the combination of these two tests was preferred during the late phase of disease progression. However, the actual sensitivity remains to be evaluated in the future.

Our results highlight that serology tests could play an important role in the diagnosis of suspected COVID-19 infections during later stage of the disease. In clinical practice, COVID-19 serological tests could contribute to the understanding of the immunological state of the population.

## Supplementary Material

Supplementary_table_2_studies_provide_direct_comparison_between_assays.docx

Supplementary_table_1_Studies_excluded_for_unreasonable_study_design..docx

Supplementary_material_2_QUADAS-2_assessment_criterion.docx

Supplementary_material_1_Search_Strategy_As_Of_30_June.docx

Supplementary_figure__PCR_sensitivity.jpg

## Data sharing

Additional data will be available on request.

## Transparency

The lead authors and manuscript’s guarantor affirm that the manuscript is an honest, accurate, and transparent account of the study being reported; that no important aspects of the study have been omitted; and that any discrepancies from the study as planned have been explained.
