# Supplementary material for: Meta-analysis of diagnostic performance of serology tests for COVID-19: impact of assay design and post-symptom-onset intervals
Source: Emerg Microbes Infect. 2020 Oct 7;9(1):2200–11. doi: 10.1080/22221751.2020.1826362 (PMC7580610; doi:10.1080/22221751.2020.1826362)
Supplement: Supplementary_table_2_studies_provide_direct_comparison_between_assays.docx [file TEMI_A_1826362_SM8056.docx]

**supplementary table 2. Studies provided direct comparison between studies**

| Study | Assay 1 | Assay 2 | Assay 3 | Assay 4 | Assay 5 | Assay 6 | Assay 7 | Assay 8 |
| --- | --- | --- | --- | --- | --- | --- | --- | --- |
| Jääskeläinen et.al 2020 | Abbott (CLIA) | Euroimmum (ELISA) | Liaison (CLIA) | Acro, USA (LFIA) | Biotime, China (LFIA) |  |  |  |
| Montensinos et.al 2020 | Euroimmum, Germany (ELISA) | Snibe, China (CLIA) | LaboOn Time, Isreal (LFIA) | Avioq, China (LFIA) | QuickZen, Belgium (LFIA) |  |  |  |
| Ong et.al 2020 | Boson (LFIA) | Cellex (LFIA) | Dynamiker (LFIA) | Orient (LFIA) | Promtheus (LFIA) | Wantai (ELISA/LFIA) |  |  |
| Traugott et.al 2020 | Euroimmum (ELISA) | Wantai, China (ELISA/LFIA) | AllTest, China (LFIA) |  |  |  |  |  |
| Tré-Hardy et.al 2020 | LIAISON (CLIA/ELISA) |  |  |  |  |  |  |  |
| Tuaillon et.al 2020 | ID.Vet, France (ELISA) | Euroimmum (ELISA) | AccuBiotech, China (LFIA) | Livzon, China (LFIA) | ISIA, China (LFIA) | UNScience, China (LFIA) | Acro, USA (LFIA) | Hecin, China (LFIA) |
| Van Elslande et.al 2020 | Euroimmum (ELISA) | Clingene (LFIA) | OrientGene (LFIA) | VivaDiag (LFIA) | Strongstep (LFIA) | Dynamiker (LFIA) | Multi-G (LFIA) | Prima (LFIA) |
