# Supplementary material for: Meta-analysis of diagnostic performance of serology tests for COVID-19: impact of assay design and post-symptom-onset intervals
Source: Emerg Microbes Infect. 2020 Oct 7;9(1):2200–11. doi: 10.1080/22221751.2020.1826362 (PMC7580610; doi:10.1080/22221751.2020.1826362)
Supplement: Supplementary_table_1_Studies_excluded_for_unreasonable_study_design..docx [file TEMI_A_1826362_SM8055.docx]

**Supplementary table 1. Studies excluded for unreasonable study design.**

| ID | Study | Exclusion Reason |
| --- | --- | --- |
| 1 | Lou et.al 2020 | 300 community natives were taken as control without confirming by RT-PCR |
| 2 | Zhao RQ et.al 2020 | using clinical-confirmed COVID-19 cases rather than NAATs-confirmed |
| 3 | Qu et.al 2020 | 394 patients confirmed with COVID-19 but only 41 enrolled without knowing why |
| 4 | Perera et.al 2020 | threshold not prespecified |
| 5 | Chen et.al 2020 | threshold not prespecified |
| 6 | Burbelo et.al 2020 | threshold not prespecified |
| 7 | Liu WB et.al, 2020 | 100 health donors not collected before 2020 neither confirmed by RT-PCR |
| 8 | Vidal-Anzardo et.al 2020 | 143 involved but only 109 tested for RT-PCR |
| 9 | Suhandynata et.al 2020 | 129 participants in control group not collected before 2020 neither confirmed by RT-PCR |
| 10 | Liu L et.al 2020 | 120 participants in control group not collected before 2020 neither confirmed by RT-PCR |
| 11 | Padoa et.al 2020 | 6 weeks from symptom onset and RT-PCT to serology detection was inappropriately too long |
| 12 | Serrano et.al 2020 | using clinical-confirmed COVID-19 cases rather than NAATs-confirmed |
| 13 | Yang et.al 2020 | threshold not prespecified |
| 14 | Hoffman et.al 2020 | convalescents patients were included; 24 health donors not collected before 2020 neither confirmed by RT-PCR |
| 15 | Kohmer et.al 2020 | participants in control group not collected before 2020 neither confirmed by RT-PCR |
| 16 | Zeng et.al 2020 | using clinical-confirmed COVID-19 cases rather than NAATs-confirmed |
| 17 | Li ZT et.al 2020 | using clinical-confirmed COVID-19 cases rather than NAATs-confirmed |
| 18 | Infantino M et.al 2020 | threshold not prespecified / partcipants in winter 2019 not confirmed with COVID-19 RT-PCR |
| 19 | Fabio C et.al 2020 | only particpants with positive LFIA test received RT-PCR for confirmation |
| 20 | Bryan et.al 2020 | threshold not prespecified |
| 21 | Du et.al 2020 | convalescents patients were included; |
