# Supplementary material for: Meta-analysis of diagnostic performance of serology tests for COVID-19: impact of assay design and post-symptom-onset intervals
Source: Emerg Microbes Infect. 2020 Oct 7;9(1):2200–11. doi: 10.1080/22221751.2020.1826362 (PMC7580610; doi:10.1080/22221751.2020.1826362)
Supplement: Supplementary_material_2_QUADAS-2_assessment_criterion.docx [file TEMI_A_1826362_SM8054.docx]

**Supplementary material 2: QUADAS-2 Tool Assessment**

**Risk of Bias**

**Domain 1: Patient Selection**

**Risk of Bias: Could the Selection of Patients Have Introduced Bias?**

1. **Was a consecutive or random sample of patients enrolled?** If articles failed to give a clear time limit or the word of “consecutive”, this question would be “No”.
2. **Was a case–control design avoided?** Only cohort studies were judged to be “yes” in this question, while all case-control and longitudinal studies were “No”.
3. **Did the study avoid inappropriate exclusions?** One study enrolled more than 300 patients but only 49 patients were tested for serology tested without explanation was rated as “No”.

**Domain 2: Index Test**

**Risk of Bias: Could the Conduct or Interpretation of the Index Test Have Introduced Bias?**

1. **Were the index test results interpreted without knowledge of the results of the reference standard?**
2. **If a threshold was used, was it prespecified?** Most studies enrolled into this meta-analysis used company kits with a clear prespecified threshold to evaluate diagnostic accuracy. Only one used self-produced protocols but determined the threshold in advance so it was “yes” in this question. (Guo L, et al. Profiling Early Humoral Response to Diagnose Novel Coronavirus Disease. Clin Infect Dis.)

**Domain 3: Reference Standard**

**Risk of Bias: Could the Reference Standard, Its Conduct, or Its Interpretation Have Introduced Bias?**

1. **Is the reference standard likely to correctly classify the target condition?** NAATs was the only method suggested by WHO in diagnosing COVID-19 and we only include studies used NAATs as a reference test.
2. **Were the reference standard results interpreted without knowledge of the results of the index test?**

**Domain 4: Flow and Timing**

**Risk of Bias: Could the Patient Flow Have Introduced Bias?**

1. **Was there an appropriate interval between the index test and reference standard?** Although interval existed between serology test and NAATs in enrolled studies, it was considered ‘appropriate’ because interval was a very important variable in our study and we kept a detailed record of it. And we did exclude some ‘inappropriate’ studies, for example, which detected serology test in convalescent COVID-19 patients.
2. **Did all patients receive the same reference standard?** COVID-19 is kind of special because it appealed in 2020. Therefore, “Yes” was set as all participants in 2020 were required for NAATs test while participants before 2020 (controls) were not required (almost all enrolled studies did like this).
3. **Were all patients included in the analysis?** For example, studies enrolled the patients but failed to collected samples from part of the patients were excluded.

**Application Concern**

**1) Applicability: Are There Concerns That the Included Patients and Setting Do Not Match the Review Question?**

a) Disease condition. Studies that only included severe or mild COVID-19 cases might lead to overestimation or underestimation of the accuracy of serology test because disease condition is closely related to immune status.

b) If participants were from a specific group, like health workers, the nursing home et.al, studies will be rated as “high concern”.

c) Studies that didn’t present a clear participant characteristic or settings were “unclear concern”

**2) Applicability: Are There Concerns That the Index Test, Its Conduct, or Its Interpretation Differ from the Review Question?**

Here we mainly evaluated the frequency of serology test. Repeating serology test on different days could overestimate the accuracy and will be rated as “high concern” in this meta-analysis. (While repeating serology test using different methods will not included because the result will not overlap)

**3) Applicability: Are There Concerns That the Target Condition as Defined by the Reference Standard Does Not Match the Question?**

Although all studies adopted NAATs as reference test, samples, different method, threshold or interpretation will also influence the applicability. Here we mainly evaluated the method for diagnosis. Studies not using RT-PCR (which is suggested by WHO) will be rated as “high concern”. (Considering that only method of NAATs could be available from most studies and our aim of evaluating is to differentiate rather than criticize)
