# Supplementary material for: Meta-analysis of diagnostic performance of serology tests for COVID-19: impact of assay design and post-symptom-onset intervals
Source: Emerg Microbes Infect. 2020 Oct 7;9(1):2200–11. doi: 10.1080/22221751.2020.1826362 (PMC7580610; doi:10.1080/22221751.2020.1826362)
Supplement: Supplementary_material_1_Search_Strategy_As_Of_30_June.docx [file TEMI_A_1826362_SM8053.docx]

**Supplementary material 1 search strategy**

1. **Key Words:**
2. COVID-19
3. SARS-CoV-2
4. Serology tests
5. Serology
6. Serologic assay
7. Antibody
8. Immunoassays
9. Sensitivity
10. Specificity
11. **PubMed:**
12. Formula: ((COVID-19) OR (severe acute respiratory syndrome coronavirus 2)) AND ((Serology tests) OR (Serology) OR (Antibodies))
13. Time limit: 1 Jan 2020 – 30 June 2020 [30 June 2020]
14. Language limit: English
15. Result: 712
16. Selected: 78
17. **Cochrane Library:**
18. Formula: COVID-19 [Title Abstract Keyword] OR severe acute respiratory syndrome coronavirus 2 [Title Abstract Keyword] AND Serology [Title Abstract Keyword] AND diagnostics [Title Abstract Keyword]
19. Time limit: 1 Jan 2020 – 30 June 2020 [30 June 2020]
20. Language limit: English
21. Result: 6
22. Selected: 0
23. **EBSCO**
24. Formula: (COVID-19 OR severe acute respiratory syndrome coronavirus 2) AND (Serology OR Antibody)
25. Time limit: 1 Jan 2020 – current [30 June 2020]
26. Language limit: English
27. Result: 549
28. Selected: 65
29. **OVID**
30. **Formula:** (COVID-19 OR severe acute respiratory syndrome coronavirus 2) AND Serology AND diagnostics
31. Time limit: 1 Jan 2020 – current [30 June 2020]
32. Language limit: English
33. Result: 609
34. Selected: 12
