# Supplementary figures and images for: Meta-analysis of diagnostic performance of serology tests for COVID-19: impact of assay design and post-symptom-onset intervals
Source: Emerg Microbes Infect. 2020 Oct 7;9(1):2200–11. doi: 10.1080/22221751.2020.1826362 (PMC7580610; doi:10.1080/22221751.2020.1826362)

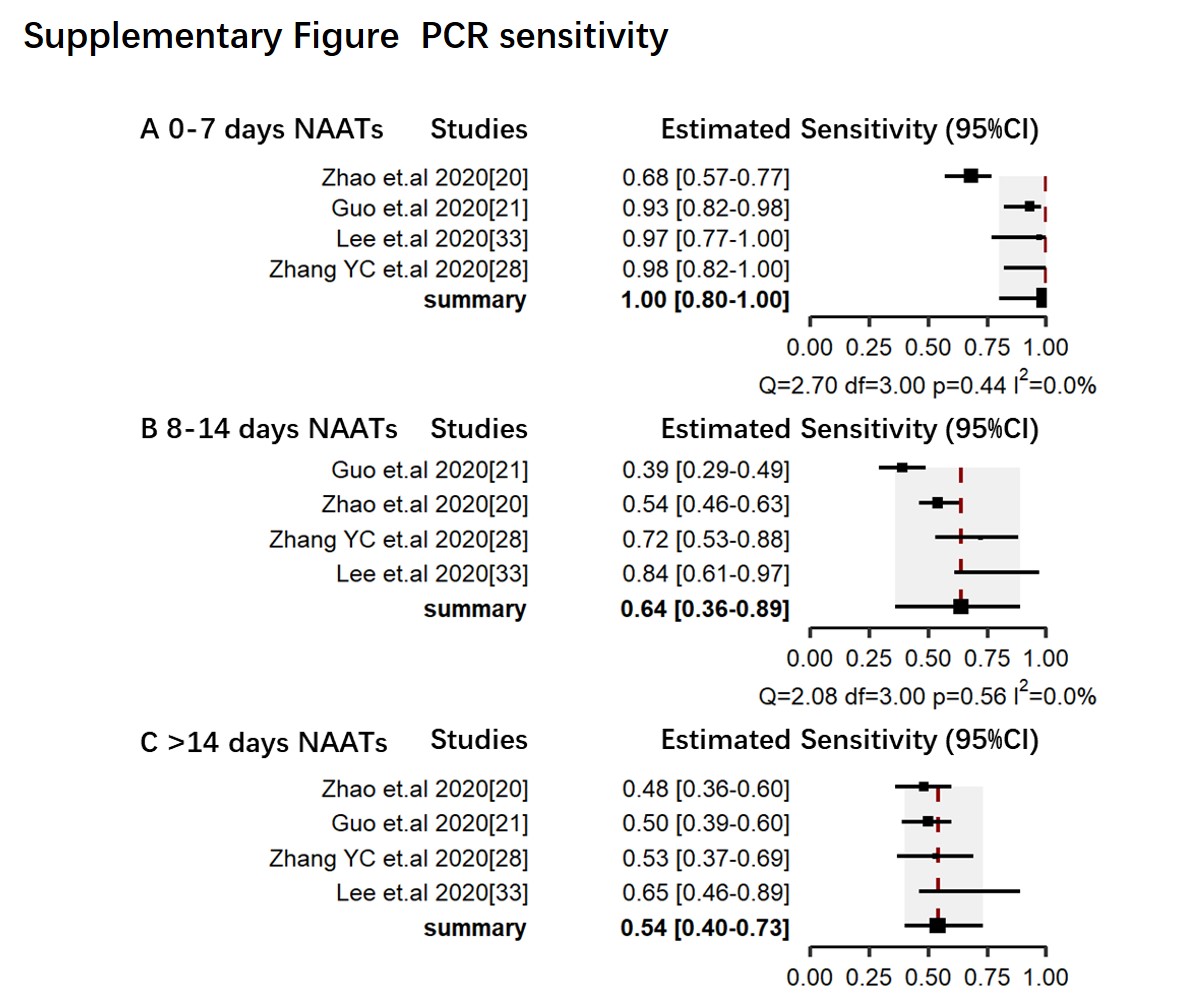

Supplement: Supplementary_figure__PCR_sensitivity.jpg [file TEMI_A_1826362_SM8052.jpg]
